# Evaluation of a hospice rapid response community service: a controlled evaluation

**DOI:** 10.1186/1472-684X-11-11

**Published:** 2012-07-30

**Authors:** Claire Butler, Laura M Holdsworth, Simon Coulton, Heather Gage

**Affiliations:** 1 Centre for Health Services Studies, George Allen Wing, Cornwallis Building, Canterbury, 56 London Road, CT2 7NF, UK; 2 Pilgrims Hospices in East Kent, University of Kent, Canterbury, CT2 8JA, UK; 3 Department of Economics, Faculty of Business, Economics and Law, University of Surrey, Staghill, Guildford, GU2 7XH, UK

**Keywords:** Rapid response service, Hospice at home, Pragmatic trial, Preferred place of death, Palliative care

## Abstract

**Background:**

While most people faced with a terminal illness would prefer to die at home, less than a third in England are enabled to do so with many dying in National Health Service hospitals. Patients are more likely to die at home if their carers receive professional support. Hospice rapid response teams, which provide specialist palliative care at home on a 24/7 on-call basis, are proposed as an effective way to help terminally ill patients die in their preferred place, usually at home. However, the effectiveness of rapid response teams has not been rigorously evaluated in terms of patient, carer and cost outcomes.

**Methods/Design:**

The study is a pragmatic quasi-experimental controlled trial. The primary outcome for the quantitative evaluation for patients is dying in their preferred place of death. Carers’ quality of life will be evaluated using postal questionnaires sent at patient intake to the hospice service and eight months later. Carers’ perceptions of care received and the patient’s death will be assessed in one to one interviews at 6 to 8 months post bereavement. Service utilisation costs including the rapid response intervention will be compared to those of usual care.

**Discussion:**

The study will contribute to the development of the evidence base on outcomes for patients and carers and costs of hospice rapid response teams operating in the community.

**Trial registration:** Current controlled trials ISRCTN32119670.

## Background

While most people faced with a terminal illness would prefer to die at home [1,2], less than a third in England are enabled to do so with many dying in National Health Service (NHS) hospitals [2,3]. Given ideal circumstances, two thirds of terminally ill people would wish to die at home [2]. Many dying patients do not have effective choice over where they die. When professional support at home is available patients are more likely to die there [4,5]. The Department of Health policy guidance [6] stresses the importance of helping patients to achieve their wishes for place of death and the potential contribution of rapid response services to this cause.

Patients with life limiting conditions are often admitted to hospital because of a crisis or challenge that could not be resolved at home [7]. The crisis often stems from uncontrolled symptoms, carer fear or stress, not having medication available, or not having enough information about the patient’s prognosis or disease trajectory [7]. Research has shown that patients who spend more time in hospital or hospice during their illness are more likely to die there [5], therefore keeping patients out of inpatient facilities may help improve the likelihood that patients will be able to die at home. Rapid response teams providing palliative care respond quickly to crises and emergencies to help patients avoid admission to hospice or hospital. They integrate with routine community care and withdraw after the crisis has resolved, which may be death. They provide intense care for a few days at a time and operate on a 24/7 on-call basis. They are available at a time when the patient and/or carer are most vulnerable and when no other service is available or able to manage the crisis.

The effectiveness of rapid response teams has not been rigorously evaluated and there have been only three studies from the UK [8-10] which were descriptive evaluations, lacking control groups and two had small sample sizes (17 patients and 62 patients). The studies all identified above national average (21%) number of patients dying at home [3], 42%, 41% and 29%, and below national average use of institutional care. Thus rapid response services appear to prevent crisis admissions and increase the number of patients able to die at home, though these evaluations lack the power to provide an adequate evidence base without a control group.

It is important to evaluate new palliative care services in terms of patients’ preferences and service delivery costs, but it is also important to consider the impact of care in terms of carers’ quality of life and experiences. The Department of Health in the *End of Life Care Strategy*[6] discusses the concept of a good death and identifies key elements of the dying persons' experience, including dignity and respect, effective pain management, familiar surroundings and presence of family and friends. However it is not clear what factors are key to carers’ experiences.

The aim of the study is to contribute to the development of the evidence base on the consequences and costs of hospice rapid response teams, compared to usual care. It will also contribute to an understanding of the ways in which carers’ perceive and evaluate a ‘good death’.

## Hypotheses

The primary hypothesis, stated as a null hypothesis, is that rapid response services will lead to similar numbers of patients dying in their preferred place of death as is achieved with usual care. Secondary hypotheses address whether the rapid response service affects the quality of life of carers and whether there are cost savings from the rapid response service compared with usual care. In addition we aim to conduct a qualitative study to explore how carers perceive the quality of care and judge a ‘good death’.

## Methods/Design

### Study design

The quantitative evaluation of the rapid response intervention is a pragmatic quasi-experimental multi-centre controlled trial, with an embedded cost evaluation. The study also includes a qualitative evaluation using in-depth interviews to explore carers’ perceptions.

### Setting

The study is based at Pilgrims Hospices in East Kent which serves a community of 600,000 through three centres based in Canterbury, Ashford and Margate. The hospices each have an inpatient ward with 16 beds, a community outreach service and a day hospice. It receives approximately 2,000 referrals each year.

### Randomisation

**Figure 1:**
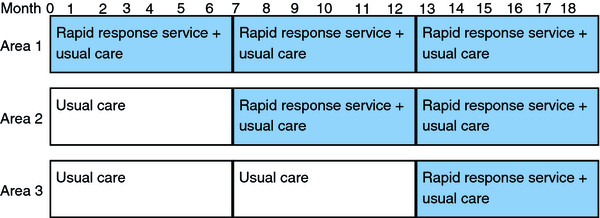
Time of service delivery by area.

The rapid response service will be rolled out sequentially in the three centres with six months between the start of provision in each site (Figure 1). A simple probabilistic randomisation method will be used to determine the order in which the three areas start the rapid response service. Once the intervention is introduced in an area, it will be available to all patients within the catchment area of that hospice, while usual services will continue to be offered in the control areas until the new service is rolled out in that area.

The evaluation design randomises centres and therefore does not require the blind randomisation of individual patients into intervention or control group which would create serious ethical and practical problems. It is a design that protects from a number of potential sources of bias: contamination between patients, changes in health policy over time and resentful demoralisation of patients [11]. The design simultaneously addresses the situational and resource factors that arise from implementing an intensive new service across a wide area.

### Intervention

The rapid response service has been developed in line with best practice and following a complete literature review of the available evidence of hospice at home and rapid response services [12]. The main features of the service are that it:

· Is available to patients in their own home (including care homes);

· Has a robust ‘hospice standard’ assessment which takes account of: patient preferences, carer/family preferences, patient needs, and patient prognosis;

· Provides hands on care;

· Responds rapidly to crises using human and material resources available 24/7 with access to health care assistants, service coordinator, palliative care nursing, medical advice, and small pieces of equipment which can be carried by car; and

· Works in coordination with other community services.

### Patient evaluation

#### Inclusion criteria

All new referrals to the hospice who are assessed by a member of the hospice team during the study period are potentially eligible for inclusion in the study and may receive the intervention if available in their area. However, as the primary outcome measure is achieving the preferred place of death only those referred who die within the intervention or control period will be included in the analysis.

#### Sample size

The primary outcome measure for the patient evaluation is death in the first recorded preferred place of death. In current 'usual' service provided by Pilgrims Hospices, approximately 29% of patients die in their preferred place of death. Increasing this figure to 60% will be equivalent to the gold standard framework for end of life care. In order to detect a difference of this magnitude, with alpha at 0.05 and 90% power, using a 2-sided test, requires data to be collected on 49 new patients per site per six month period. The design of the study involves 9 cells (Figure 1) so the total sample size is inflated to 441. This equates to 147 for each of the three sites and 147 for each six monthly period.

#### Clinical outcomes

Preferences and any changes in preference will be ascertained by hospice nurses or doctors undertaking community, inpatient or clinic based assessments. Actual place of death will also be recorded.

#### Data collection

**Figure 2:**
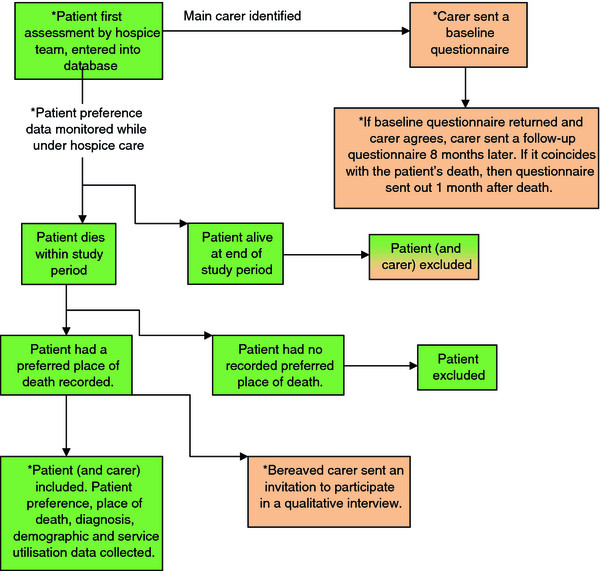
Patient and carer data collection flow chart for both intervention and control groups, * indicates data collection points.

Data collection for the patient outcome will be conducted in a two stage process (Figure 2). First, patients will be entered onto a database after they have had their first assessment with a member of the hospice team. It is at this point that carers will be sent a questionnaire. Patient preferences will then be monitored while under hospice care in order to identify any changes in preferences over time. At the end of the study period, any patients still alive will be excluded as well as their carer. Patients who have died during the study period and who had a preferred place of death recorded in the hospice notes will be included. At this point, these patients’ preference data, place of death, diagnosis, demographic data and service utilisation data will be extracted from service records. Patients who died during the study period but did not have a preferred place of death recorded will be excluded from analysis, but carers of this group will be included.

#### Data analysis

The main hypothesis will be tested using logistic regression controlling for baseline values and cluster using robust standard errors. Achieving preferred place of death will be presented as odds ratios, for control and intervention periods with associated 95% confidence intervals. A secondary analysis will explore last stated preferred place of death versus actual place of death using a similar approach.

#### Economic evaluation for patients

A critical aspect of an evaluation such as this is to include a measure of impact from an economic perspective particularly one that could be utilised by commissioners to make decisions regarding the relative economic impact of an intervention. The costs of the intervention will be calculated on an individual patient basis. Staff time input, mileage travelled to patient homes and consumables used in the delivery of the rapid response intervention will be obtained from the service’s activity logs and patient records. Resource utilisation will be converted to costs using nationally validated unit costs, for staff time [13], and information from local financial managers for expenditure on travel and consumable items. The overall service utilisation of all participants during the time they are in the study will be collected for all patients in both arms including: general practice and community resources, outpatient, inpatient stays, out of hours service, Marie Curie nurse visits, and social care packages. This information will be gathered from providers’ databases for the period that each patient is in the study, and converted to unit costs [13]. The extent to which the rapid response service substitutes for other forms of health and social care (both community-based and inpatient) will be assessed.

The difference between the mean cost of patients in the rapid response arm and patients receiving treatment as usual will be calculated and compared with the difference between groups in the primary effectiveness outcome (proportion dying in preferred place of death). If the hospice intervention results in significantly improved outcomes at lower cost, it becomes the service delivery option of choice. If the intervention achieves superior outcomes (significantly more patients dying in their preferred place), but at higher cost, the cost per percentage point gain in dying in the preferred place will be calculated.

### Carer evaluation

Carers will be included in the study if they cared for a patient who died within the study period. Only one main carer per patient will be sampled.

#### Sample size

The primary outcome measure for the carer evaluation is quality of life, measured using SF12 [14], at patient intake to hospice services and 8 months later. A clinically important change for quality of life is estimated as a 5 point change on the SF12. This equates to a medium effect size difference between the groups of 0.5. In order to establish an effect size difference of this magnitude at 80% power, with alpha at 0.05 requires 56 people within each of the intervention and control cells of the study. Previous experience with similar populations suggests 50% will refuse consent and the follow up rate at 8 months will be of the order of 70%. This inflates the required sample of potential carers to 160 in each cell of the study design, a total of 1440. Assuming that 40% of end of life patients have no primary carer the numbers seen over the study period based on current referral rates allow for a potential recruitment population of 1800.

#### Outcomes for carers

Carer outcomes will be measured at baseline on patient intake and 8 months later using self completion postal questionnaires. The primary outcome measure will be the short-form SF12 [14]. Other outcomes include a measure of anxiety and depression (HADS) [15], a measure of health utility (EQ-5D) [16], and caregiving demand [17] measured at baseline only. In the follow-up questionnaire a satisfaction with care questionnaire will be included.

#### Data analysis

Prior to the analysis of outcomes distributional assumptions will be checked and the analytical framework adjusted to account for these. Analysis of quality of life will be conducted using analysis of covariance, or non-parametric equivalent, adjusting for baseline values and carer burden. Potential cluster effects will be explored using robust standard errors. A similar approach will be employed for other carer outcomes. Results will be presented as means per group and appropriate estimates of precision.

### Qualitative exploration of carers’ perceptions

In our review of the evidence we were unable to locate an established and agreed way of assessing carers’ perception of a good death and the experiences of the dying process. This study provides a unique opportunity to explore this issue with carer respondents.

#### Sample and access

Approximately 6 to 8 months after death invitations to participate in an interview will be sent to select bereaved carers of patients who expressed a preferred place of death. Given the exploratory nature of these interviews, a purposive sample is appropriate. In order to include a range of views, we will aim to recruit up to 60 bereaved carers from both intervention and control arms and carers of patients who both achieved their preferred place of death and those who did not.

#### Interviews

Interviews will be conducted using a semi-structured topic guide which will allow the researcher to cover a range of topic areas but with some flexibility so that the respondent is free to discuss and explain their own views, experiences and feelings [18]. The intention is to elicit complex, in-depth data in relation to end of life care. Interviews will take place at a time and place convenient to the respondent and will be designed to last up to an hour.

#### Data analysis

Interviews will be recorded (if the respondent agrees) and transcribed. Analysis will follow the framework method [19], which is a matrix based thematic analytic method in which the links between the ‘raw’ data and different levels of abstraction are maintained through transparent data management. A purposive sample of this nature will enable a thematic analysis and identify the range of respondent experiences. There should also be ample data to undertake a comparative analysis for the intervention and control group carers, and between those whose relatives died in their preferred setting and those who did not. Analytical rigour will be maintained by using a second researcher to code a proportion of the transcripts to test for reliability within the coding framework.

## Ethical approval

Ethical approval was granted by Kent Research Ethics Committee, reference 09/H1101/75. The study will be conducted in accordance with the Helsinki declaration.

## Discussion

The collection and recording of the patient’s preferred place of death was regarded as a routine clinical activity. Early in the study, an intervention designed to assist and encourage staff with this data collection was planned through meetings between the research team and clinical staff.

The research has been funded by the National Institute for Health Research through the Research for Patient Benefit funding stream. The clinical service intervention was funded by Eastern and Coastal Kent Primary Care Trust (PCT) for the duration of the study. During the setting up of the study, there were conflicts in terms of timescales between the PCT’s requirements for clinical service development and the requirements of the research project, such as allowing time for ethics and governance approvals and a baseline data collection period for all three sites.

## Competing interests

The authors declare they have no competing interests.

## Authors’ contributions

CB made substantial contributions to conception, design and interpretation of data for the study. LH contributed to the study design, data collection and analysis. SC contributed to the overall study design and the quantitative analysis aspects of the study. HG is responsible for the economic analysis. All authors contributed to drafting and approved the final manuscript.

## Pre-publication history

The pre-publication history for this paper can be accessed here:

http://www.biomedcentral.com/1472-684X/11/11/prepub
